# Picking the right piece: Action intentions shape visual search and action planning in human multi-target-foraging

**DOI:** 10.1016/j.isci.2025.112656

**Published:** 2025-05-13

**Authors:** Danilo A. Kuhn, Jan Tünnermann, Anna Schubö

**Affiliations:** 1 Department of Psychology, Philipps-University Marburg, 35032 Marburg, Germany

**Keywords:** Natural sciences, Biological sciences, Neuroscience, Clinical neuroscience, Social sciences, Psychology

## Abstract

Everyday behavior, such as grocery shopping, involves searching for multiple similar objects (“visual foraging”). Although object search is usually performed in order to interact with the object, only a few studies used real objects. In object interaction, the interaction type and precision requirements likely affect object selection. When high precision is required, actions are performed more carefully (e.g., with lower speed). To investigate action context in interactive real-world multi-target foraging, we asked participants to pick and place LEGO bricks and varied the precision requirements with different placing instructions. Movement analysis revealed that participants preferred nearby objects but also prioritized those beneficial to the task, such as larger objects, when creating a pile. This demonstrates that participants planned reach movements by balancing immediate movement costs with future precision demands. In sum, task and action context, such as placing requirements or the environmental layout, must be considered for understanding visual selection in real-world situations.

## Highlights

• In multi-target foraging, objects are selected to minimize motor and search efforts
• Spatial search organization seems highly tuned toward the particular action task
• Selection strategies adapt to biomechanics depending on the environment and context
• Foraging tasks can reveal the dynamical interplay of action and perception

## Graphical abstract

**Figure:**
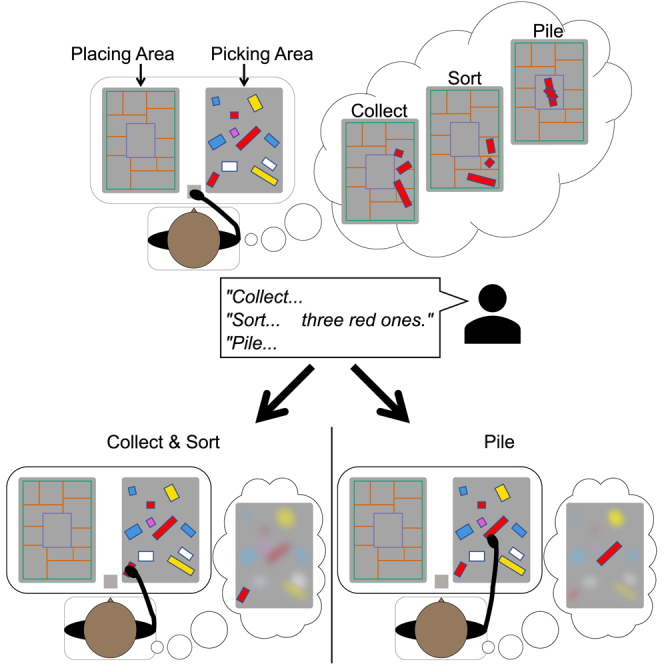

## Introduction

Reaching for an object to subsequently perform an action with it is a routine for humans. Such actions can be diverse: Putting a cup in the dishwasher requires precise placement among other objects, whereas putting a cup in the empty sink requires less precise placement. Such differences in precision requirements when placing the same object influence our reach-to-grasp movement. Prior studies showed that planning an action that requires more precision (e.g., placing in a narrowly defined area vs. placing without spatial restrictions) leads to longer reach movement times, lower reach movement speed, and a longer deceleration period.^1^^,^^2^^,^^3^^,^^4^^,^^5^ This is usually interpreted to result from a more careful object approach in order to grasp the object more precisely in preparation for the increased precision requirements when placing.

Bekkering and Neggers^6^ examined whether different action intentions would also influence visual selection. Participants either grasped or pointed to a target defined by color and object orientation. The results showed that participants made fewer initial saccades to distractors that differed in their orientation from the target when grasping than when pointing to it. Because target orientation is a relevant dimension for grasp planning but not for pointing, this finding indicates that action intentions modulate visual selection. Further studies supported this notion, suggesting that during early visual processing, different intentional weights are set for feature dimensions, depending on their relevance for an upcoming action.^7^^,^^8^^,^^9^^,^^10^^,^^11^

**Figure 1:**
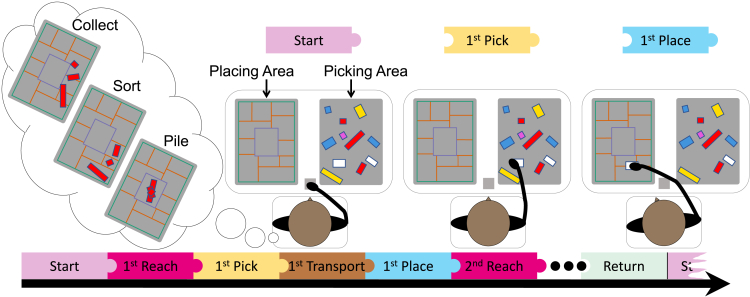
Basic setup and placing instructions In each trial, participants were asked to sequentially pick and place a fixed number of LEGO bricks defined by color (see Video S1 for a sample trial). Placing instructions varied between trials (collect, sort, pile). For analysis, movements were segmented into reach and transport movements. The first reach was defined as the movement from the starting position (pink) to the first target in the picking area (yellow). After grasping and picking up an object, the transport movement started, which ended when the object was placed in the placing area (blue). There, the second reach movement started back to the picking area. Please refer to the online version to view the figure in color.

While these studies showed that action intentions influence selective visual attention in simple reach-to-grasp or reach-to-point movements, it remains open whether action intentions also influence (visual) selection in more natural scenarios that include multiple targets^12^^,^^13^^,^^14^^,^^15^^,^^16^ and movement sequences.^17^^,^^18^^,^^19^ Using visual foraging-like action sequences, we investigated the impact of action intention,^6^ also studied in isolated actions as “movement intent,”^3^ “prior intention,”^20^^,^^21^ or “second-order (motor) planning”^22^ on target selection in naturalistic search. In our foraging experiment with 3D objects, participants picked and placed an instructed number of LEGO bricks (search sizes: 3, 4, 5) defined by color. Each trial initially contained six targets, so participants never had to search exhaustively, but could choose which targets to select or avoid.^14^ We varied the precision requirements using different placing instructions (collect, sort, pile, see Figure 1; a sample trial can be seen in Video S1). Tracking accuracy of the markerless system was validated by visual inspection and a low test error of 4.73 pixels (about 0.2 cm; see pose estimation for details). Movements were segmented into *reach phases* (the movement toward a target in the picking area)^3^^,^^23^ and *transport* phases (movement with the object from the picking to the placing area).^2^^,^^24^ To this end, different sources of information were combined to find optimal segmentation points.^25^ To detect the start of a reach movement, for example, the hand position was required to be at the start key for picking the first target or in the placing area for picking all subsequent targets. Further, speed minima were factored into the movement segmentation to ensure that it was the beginning or end of a movement. Speed had to exceed 30 cm/frame to guarantee that a movement had actually started. Moreover, an acceleration threshold of 0.5 cm/frames^2^ allowed us to remove artifacts (see data preprocessing for details). From the final dataset of 24 participants (1944 trials), 251 trials were removed from reach analysis and 159 trials from transport analysis because these trials could not be properly segmented by the algorithm (e.g., if a target was not placed but dropped at high speed, the transport and subsequent reach movement could not be separated). Last, incorrectly segmented trials were removed after final visual inspection (reach analysis: 5 trials, transport analysis: 3 trials). 46 trials had to be removed due to incorrect responses by participants (e.g., picking the wrong number of bricks), and 5 trials due to technical failures in recording. 1637 trials (84.2%) remained in the dataset and were submitted to the reach movement analysis, while 1731 (89%) remained for transport movement analysis. In each trial, participants started the movement sequence at a predefined starting position in front of them before they performed the instructed number of reaches and transports to complete the trial. In the placing area, they placed the items according to the pile, sort, or collect instructions (varied between trials) as shown in Figure 1. When sorting, participants were asked to place the targets in any separate fields, but were not instructed to sort the targets by size or shape. After each trial, the experimenter put the previously selected items back in the picking area. Following each block of nine trials, the experimenter distributed all LEGO bricks at random in the picking area using a 5 × 8 3D grating, which was removed before trial start.

Video S1. Sample trial in the pile condition, related to Figure 1 A participant sequentially picks and places four red LEGO bricks in the pile condition.

## Results and discussion

### Placing instructions influence object selection, planning, and action execution

When analyzing how participants interacted with this physical task, we found that participants had the longest deceleration phase when transporting the items for piling (*M* = 54.7%, 95% CI [53.7, 55.6]), followed by sorting (*M* = 53.5%, 95% CI [52.5, 54.5]) and placing (*M* = 51%, 95% CI [49.7, 52.3]). The Bayes factor [BF_10, U_] quantifies how much more likely the data are under an alternative model, which assumes a difference, compared to the null model, which assumes no difference (for more details, please refer to the General Statistical Analysis), and was 4.9 for piling compared to sorting and 183 for sorting compared to collecting. This provides evidence that participants took the instructions into account and adjusted their actions toward them, placing objects more carefully with higher precision demands, in line with earlier studies.^1^^,^^2^^,^^3^ Some studies^2^^,^^3^^,^^4^ reported that task goals with higher precision requirements lead to more careful approaches when picking up objects, indicated by a longer deceleration phase. Interestingly, we did not observe this effect. Instead, we found that the relative time spent decelerating was equally long under all instructions (collecting: 49.9%, 95% CI [49, 50.8], sorting: 50.2%, 95% CI [49.4, 51], piling: 49.9%, 95% CI [49, 50.8]; BF_excl_ = 8.4; see statistical analysis of first reach deceleration time (%) for the full ANOVA table; quantification and statistical analysis contains all interactions for which there were no hypotheses in order not to overload the main text). Several reasons might have contributed to this pattern: As shown in a meta-analysis,^1^ larger differences in the precision requirements between two upcoming tasks (e.g., throw vs. place) lead to larger differences in reach deceleration time. Perhaps our placing instructions were too similar in difficulty to elicit differences in deceleration duration. Moreover, in previous studies that showed an increased deceleration duration for more difficult tasks, participants picked and placed a single, isolated object. In our task, participants searched and collected several objects in a trial, always selecting targets among other objects. This way, the objects surrounding a target may have served as obstacles, which required participants to decelerate their movements to a similar degree in all placing instructions in order to adjust contact points for grasping.^26^

**Figure 2:**
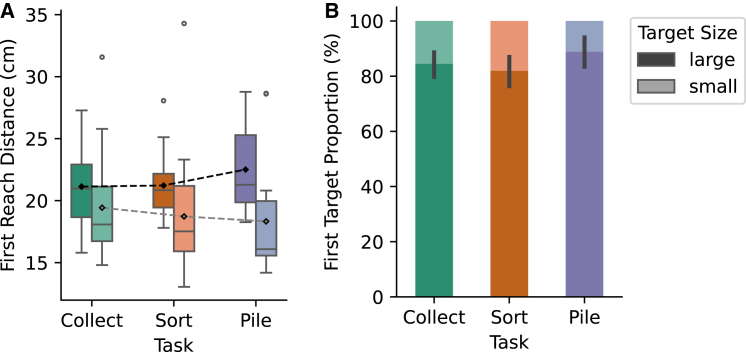
Effects of placing instructions and selected target size on first reach movement (A) Average first reach distance (y axis) per placing instruction (green: collect, orange: sort, purple: pile; x axis) when large items (dark) or small items (light) were selected. Black diamonds represent the mean value of the respective task goal. The box plots show how the data points are distributed. For this purpose, the quartiles were calculated. The central box ranges from the first quartile to the third quartile and thus shows the interquartile range (IQR), which includes 50% of the data. The central horizontal line corresponds to the median, with 50% of the data lying above or below this value. The whiskers extend to the last data point, which is within 1.5 times the IQR. Data outside this range are outliers. (B) Average proportion of the first target being of large size (dark) or small size (light; y axis) per placing instruction (green: collect, orange: sort, purple: pile; x axis). Error bars denote the standard error of the mean. Please refer to the online version to view the figure in color.

Previous studies also showed that higher task difficulty leads to a decreased reach speed.^1^^,^^3^ Interestingly, in our study the first reaching movement was the fastest (50.3 cm/s, 95% CI [47.5, 53] on average over the whole trajectory) in the most difficult task, the piling condition (compared to 47.9 cm/s, 95% CI [45.6, 50.2] in sorting [BF_10, U_ = 113.4] and 48.3 cm/s, 95% CI [46.2, 50.5] in collecting [BF_10, U_ = 3.5]). Note that only the first reaching movements started from the same position in all trials; the other reaches are more difficult to compare between the placing instructions. The faster speed in the piling condition was accompanied by a longer average reaching distance of 22.2 cm (95% CI [20.8, 23.7]) for piling compared to 20.9 cm (95% CI [19.7, 22.2], BF_10, U_ = 35.8) and 21 cm (95% CI [19.8, 22.3], BF_10, U_ = 3.4) for sorting and collecting. There was, however, some variability in how participants interpreted how to pile objects, which is discussed in a later section. Nonetheless, participants might have accepted larger distances when piling to select targets that are particularly useful in the action sequence. Selecting large items early in the sequence and small items later facilitates the overall piling process and thus task completion. To check this, we performed directed *t*-tests, expecting the first reach distance to be longer for large items when piling. Indeed, average first reach distance for large items was highest in the piling condition (*M* = 22.5 cm, 95% CI [21.2, 23.8]) compared to collecting (*M* = 21.1 cm, 95% CI [19.9, 22.3]; BF_+0_ = 7.8) and sorting (*M* = 21.2 cm, 95% CI [20.2, 22.3]; BF_+0_ = 13.2) as shown in Figure 2A. The Bayes factors indicate that the data are 7.8 (13.2) times more likely under a model assuming a longer first reach distance for larger targets when piling compared to collecting (sorting) than under the null model that assumes no difference. To check whether this effect was specific for large items, we performed directed *t*-tests because we expected that the first reach distance was also longer for small items when piling. For small items, however, there was evidence against a larger first reach distance in the piling condition (*M* = 18.3 cm, 95% CI [15.8, 20.9]) compared to collecting (*M* = 19.4 cm, 95% CI [17.7, 21.1]; BF_0+_ = 8.8) or sorting (*M* = 18.7 cm, 95% CI [16.9, 20.6]; BF_+0_ = 6.2). The faster reach speed shows that participants adjust their movement parameters to compensate for the longer distances, perhaps to maintain a stable rhythm in the oscillating pick-and-place actions. This seems to agree with findings that discrete actions can merge into stable rhythmic patterns even though the task does not require periodicity.^27^

Further tests revealed decisive evidence that participants tended to select larger objects almost six times as often as smaller objects with their first reaching movement (Figure 2B; *M* > 80%, 95% CI between [76.2, 87.7] and [83.1, 94.5] for all placing instructions, BF_10_ between 6.157 × 10^6^ and 1.457 × 10^8^ on the arcsine transformed proportions that they are different from 50%). This is in line with earlier studies^28^^,^^29^^,^^30^ and seems to reflect, to some degree, attentional preferences.^31^ Selecting a large item first would be more advantageous when piling because it could serve as a more stable foundation. We performed directed *t*-tests to check whether the preference for a first large item was stronger when piling. Indeed, this effect was the strongest for piling with 88.8% (95% CI [83.4, 94.2]) compared to 84.4% when collecting (95% CI [79.9, 89]; BF_+0_ = 6.1) and 81.9% when sorting (95% CI [76.5, 87.3]; BF_+0_ = 34.9).

In sum, results show that precision requirements induced by placing instructions influence which target objects are selected, and that action sequences are planned with regard to the action intention. Selective visual attention presumably fostered the dynamical prioritization of potential targets in the action sequence.^32^^,^^33^ Theories of selective attention assume that priority is, among other factors, jointly generated from stimulus-driven and goal-directed guidance.^34^^,^^35^^,^^36^ Stimulus-driven factors might have boosted some object locations more than others, whereas prioritization according to task demands (e.g., finding an object of particular size) was accomplished by goal-driven factors that ensured picking the right targets at the right time.

### Spatial selection patterns

**Figure 3:**
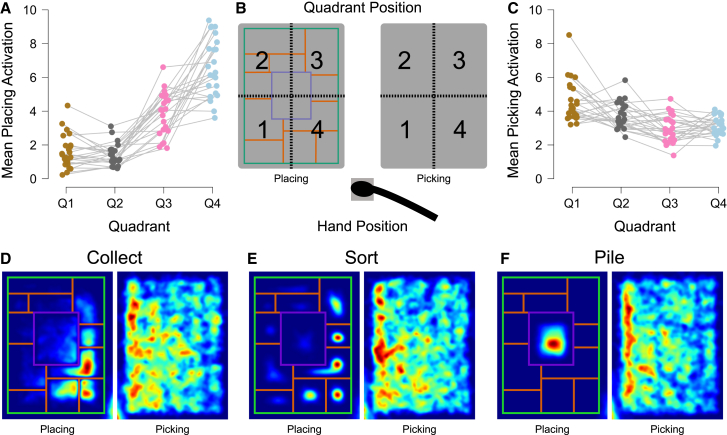
Strategy use for picking and placing (A) Mean heatmap activation (y axis) quantified by placing subareas 1–4 as shown in (B) across placing instructions (collect, sort, pile). (B) Division of picking area (left) and placing area (right) into four quadrants for the quantification of spatial preferences. (C) Mean heatmap activation (y axis) quantified by picking subareas 1–4 as shown in (B) across placing instructions (collect, sort, pile). (D–F) Heatmaps show the activation of local placing preference (always left) and local picking preference (always right) for separate task instructions across all participants. Brighter and red colors indicate a larger activation, and dark blue indicates no activation. In (A) and (C) each dot represents the mean of a participant. Please refer to the online version to view the figure in color.

Studies on visual search showed that participants aim to minimize efforts and maximize performance, for instance by adjusting their attentional control strategies to select beneficial targets^37^^,^^38^^,^^39^^,^^40^ or nearby items.^28^^,^^41^^,^^42^^,^^43^ Similarly, studies on motor control showed that participants choose movements with lower motor costs (in terms of biomechanics, force and duration).^44^^,^^45^^,^^46^ As in our paradigm, both attention-related and action-related costs combine, we expected such optimizations to be reflected in the spatial selection patterns, given that our task left participants sufficient freedom by not requiring them to act on all present targets. Figures 3D–3F show a quantification of where participants interacted with the place and picking areas. We found that participants preferably selected targets on the left side, with a stronger bias to the lower left quadrant (see picking areas in Figures 3D–3F). Also, participants preferably placed items on the right side, with a larger bias to the lower right quadrant (see placing areas in Figures 3D–3F). The activity distributions are constrained by the allowed placing areas. In the picking area, where participants had more freedom concerning where to act, the activity is more dispersed. For statistical treatment, we followed Woods et al.,^47^ and divided the picking area into four equally sized quadrants. A Bayesian repeated measures ANOVA with the factors quadrant (1, 2, 3, 4; see Figure 3B), task (Collect, Sort, Pile), and search size (3, 4, 5) revealed decisive evidence for a main effect of quadrant on picking preference (BF_incl_ = 4.666 × 10^6^) and search size (BF_incl_ = 7.740 × 10^11^; see statistical analysis of picking activation for full table), showing that the observed data are several orders of magnitude more likely under the alternative model, which assumes an effect, compared to the null model, which assumes no difference. Post hoc tests showed that the lower left quadrant contained the most activity (lower left quadrant: *M* = 1.8, 95% CI [1.6, 2]; compared to the other quadrants with *M* and 95% CI between 1.1 [1, 1.3] and 1.4 [1.3, 1.6]; BFs_10, U_ > 14014; see Figure 3C) and that activation increased with search size (3 targets: *M* = 1.1, 95% CI [1.1, 1.2]; 4 targets: *M* = 1.4, 95% CI [1.3, 1.5]; 5 targets: *M* = 1.6, 95% CI [1.5, 1.7]; BFs_10, U_ > 119931).

In sum, participants (all right-handed) preferred selecting targets near the placing area and closer to themselves and the starting position, possibly minimizing the effort incurred by their selections. There are several factors that might have contributed to the bias for the near-left quadrant. Previous studies suggested that attentional processing is facilitated for objects with high-affordance character in near-hand or action-space regions,^48^^,^^49^^,^^50^ which may have strengthened the preference for targets in the near-left quadrant. In a 2D visual search task, participants exhibited a search bias to the left side, while also preferring to initiate search in the upper half, analogue to western reading habits.^47^ In a 3D virtual reality study, however, participants exhibited a bias to initiate search in the lower visual field which contained objects closest to them,^51^ suggesting that even a habitual search strategy would allow some flexibility in where the search is initiated. Further, a natural arm comfort zone might play a role. Selecting a target in the near-left quadrant allows an elbow rotation that is close to the middle of the range of motion, allowing more elbow rotation flexibility for upcoming movements.^52^ Arguably, task ergonomics, such as the location of the placing area, likewise might have contributed to the pattern. In our spatial layout, the near-left quadrant was the quadrant closest to the start position, and together with the far-left quadrant, the one closest to the placing area. By selecting targets from the near-left quadrant, both reach and transport distances could be minimized. Also, by selecting items on the left side, participants could minimize effort by avoiding long movements away from the body center.^45^ In addition to minimizing action efforts, spatial patterns might also structure the visual search. When targets are repeatedly found in a prioritized area, attention might be guided to this area in repeated searches.^53^^,^^54^ Presumably, attentional and motoric prioritizations can align and mutually enhance each other in such tasks.

### Further observations and outlook

Offering participants a more flexible task than in many other experimental paradigms enables a greater variability in behavior that can be challenging to quantify or might interact with experimental factors. We observed that participants interpreted the piling conditions quite variably. We informally identified three behavioral patterns, with high consistency within participants. Ten out of 24 participants placed the targets in the central piling area without piling them there, making it more similar to the collect condition, but in a smaller area. The remaining 14 participants stacked the LEGO bricks on top of each other or put them on top of each other without stacking them. Placing targets in the piling area without piling them there might mean that those participants did not search for a specific item (e.g., not the largest), thereby reducing the observed effect that large objects were most frequently selected in the piling condition (Figure 2B). Additionally, this increase in variability in the pile condition may hide other, smaller effects of task difficulty on movement parameters, such as reach duration or movement onset. Repeating the same experiment with more constrained task instructions in the piling condition could shed light on this.

For those 14 participants who put the targets on top of each other (with or without stacking the bricks together), we further made a few informal observations that seem to support the idea that longer-term planning is included in their foraging behavior. Participants’ tendency to select a large item decreased over selections. As a second target, participants still appeared to prefer large items, despite their reduced availability. Search size (i.e., how many targets participants had to forage) seemed to modulate the further target choice: For search size three, participants appeared to prefer a nearby item as the third (and last) target. This target was now likely to be small if participants had previously selected two of the three larger items. For search sizes four and five, however, a preference for small items only appeared with the fourth target selection. Participants now also appeared to accept larger reach distances for especially small items, opposite to their behavior at trial start. Additionally, such longer-term planning may differ between individuals: While some participants exhibited selection patterns as described above, others appeared to give less consideration to target size.

Another point in which our paradigm might be expanded is in the stimulus presentation. We expected to see effects of placing instructions on movement onsets, which did not emerge. One potential reason is that participants could see the search scene before a trial started while listening to the search instructions, which were about 2 s long. During this time, participants might have already started visually selecting the potential targets and planning their movements. If this were the case, a later onset of vision availability should elicit a difference in movement onset between the placing instructions. Alternatively, no difference in movement onset could suggest sequential movement planning.^55^ However, this would contrast with the finding that participants plan several targets ahead when foraging in 2D.^28^

Generally, it is very likely that participants used this preview to preplan their search at least to some degree. In the present study, we lack a no-preview baseline to quantify this influence. Initial reaction times do not seem particularly fast (*M* = 562.6 ms), but this is difficult to judge without a baseline. As a tentative approach, we looked at the deviation from the straight line: We calculated the reach path ratio or reach path efficiency by dividing the traveled path by the direct path. It provided a mean of 1.12, which is close to the ideal path ratio of 1, and similar to a reach-to-grasp study that allowed preplanning and reported a reach path ratio of 1.08.^56^ Therefore, it seems plausible that searchers used the preview for preplanning, but performing a definite test remains a goal for future work.

### Conclusions

Our findings demonstrate that in natural settings, target selection and reach movement planning depend on goal-related factors such as precision requirements when placing objects. Targets are selected to minimize action (and possibly also attentional search) efforts, but are biased based on their immediate usefulness for the task goal. The spatial search organization seemed highly tuned toward the particular task. Taken together, our results highlight that understanding allegedly *visual* search in more realistic scenarios needs to take also the *motoric* actors into account.

### Limitations of the study

Limitations of the study can be seen in the variability among participants in the interpretation of how to pile objects, and in the potential impact of the preview time on movement planning. Both points are discussed in the section further observations and outlook.

## Resource availability

### Lead contact

Further information and requests for resources should be directed to and will be fulfilled by the lead contact, Danilo A. Kuhn (danilo.kuhn@uni-marburg.de).

### Materials availability

No new materials were generated in this study.

### Data and code availability

• Raw and preprocessed data have been deposited at the Open Science Framework and are publicly available as of the date of publication at https://doi.org/10.17605/OSF.IO/C938E.
• All original code has been deposited at the Open Science Framework and is publicly available at https://doi.org/10.17605/OSF.IO/C938E as of the date of publication.
• Any additional information required to reanalyze the data reported in this article is available from the lead contact upon request.

## Acknowledgments

This research was supported by “The Adaptive Mind,” funded by the Excellence Program of the Hessian Ministry of Higher Education, Science, Research and Art. This work was further supported by the 10.13039/501100001659Deutsche Forschungsgemeinschaft (DFG, German Research Foundation – SFB/TRR 135, project number 222641018, TP B3). We thank Carola Breitmaier and Julia Elina Stocker for their assistance with data collection.

## Author contributions

Initials in alphabetical order; conceptualization: A.S., D.A.K., and J.T.; stimulus and task development: A.S., D.A.K., and J.T.; data acquisition: D.A.K.; formal analysis and modeling: D.A.K. and J.T.; writing (original draft): D.A.K.; writing (reviewing and editing): A.S., D.A.K., and J.T.; supervision: A.S., J.T.

## Declaration of interests

The authors declare no competing interests.

## STAR★Methods

### Key resources table

**Table:** 

REAGENT or RESOURCE	SOURCE	IDENTIFIER
**Deposited data**		

Raw and preprocessed data.	This paper	https://doi.org/10.17605/OSF.IO/C938E

**Software and algorithms**		

OpenSesame	Mathôt et al.^57^	https://osdoc.cogsci.nl/
DeepLabCut	Mathis et al.^58^	https://github.com/deeplabcut
JASP	JASP Team^59^	https://jasp-stats.org/
Python version 3.7 and 3.8	Python Software Foundation	https://www.python.org
SciPy version 1.7	Virtanen et al.^60^	https://scipy.org/
DeepLabCut-project script and python code for data analysis.	This paper	https://doi.org/10.17605/OSF.IO/C938E

**Other**		

DFK 37AUX273	The Imaging Source	https://www.theimagingsource.com/en-de/product/industrial/37u/dfk37aux273/

### Experimental model and study participant details

Thirty healthy human volunteers originally participated in the study. Foraging tasks are fast-paced and include many individual reactions. They typically provide sufficient statistical power with moderate participant numbers. Studies looking at effects on target choice found robust effects with as few as 16 participants^13^ and fine-grained effects on the collecting action were demonstrated with 20 participants.^16^ Given the additional flexibility encountered in real-word contexts, we recruited 30 participants. Data of six participants were not considered due to technical issues during movement segmentation (see Data Preprocessing for details). After data preprocessing, 24 participants between 18 and 36 years of age remained for further analyses (M = 25.4 years, SD = 4.6; 14 females; 20 right-handed and four mixed-handed with a mean laterality quotient of 76.9, SD = 22.4). Handedness was assessed using a brief form of the Edinburgh Handedness Inventory.^61^^,^^62^ All participants reported correct or corrected-to-normal vision and no severe color deficiencies. Experience with handling LEGO® bricks was assessed with a scale between 1 (no experience) and 5 (a lot of experience). The mean of the participants’ experience rating was 2.4 (SD = 1). Ethnicity was not recorded.

All participants were informed about the aims of the study as well as about the storage and processing of their anonymized data and gave their written consent. Participants received either course credit or financial compensation (8 €/h) and were naïve as to the purpose of the study. The experiment complied with the 1964 Declaration of Helsinki and the approvals of the Ethics Committee of the Department of Psychology at the Philipps University of Marburg (protocol number: 2022-35k).

### Method details

#### Apparatus and materials

Participants were seated in front of a table with a height of 74.5 cm. On the table, two grey pads were positioned, serving as the picking area, where stimuli were presented (right), and the placing area (left). The two pads had a size of 22 × 30 cm, with the short side facing the participant. The distance from the edge of the table to the pads was 5.5 cm, and the distance between both pads was 16 cm. A single-key USB keyboard, sized 3 × 3 cm and approximately 3 cm in height, served as the start and end point for each trial. The key was located between both pads, 3.5 cm from the edge of the table on the centerline of the participants. The basic experimental setup is displayed in Figure 1.

An industrial camera manufactured by The Imaging Source was used to record with 200 frames per second at full resolution of 1,440 × 1,080 pixels. To receive footage from a bird’s eye view, a USB 3.1 color camera (The Imaging Source, DFK 37AUX273) with a 1/2.9-inch Sony CMOS Pregius sensor (IMX273LQR-C) was centrally mounted 175 cm above the table. The camera was connected to a workstation with Windows 10 Education and an Intel Core i9-9820X CPU with a clock speed of 3.3 GHz. OpenSesame^57^ (Version 3.3.11) with Python 3.7.6 was used for running the experiment and trial-wise video recording with IC Imaging Control .NET Library (Version 3.5.6; The Imaging Source, 2020).

40 LEGO® bricks were positioned in the picking area, including six of each possible target color (blue, red, yellow) with various shapes (see LEGO® Brick Name (Design ID) of Targets by Size and Target Set). Between participants, these 18 items differed in color-shape combinations but not in the total color or shape count. The remaining 22 items (Table S1) were of distinct colors across all participants.

**Table:** LEGO® Brick Name (Design ID) of Targets by Size and Target Set

Classified Size	Target Set a	Target Set b	Target Set c
Small	1 × 1 (3005)	1 × 1 (3005)	1 × 2/45° (3040)
	1 × 2/45° (3040)	1 × 2 (3004)	1 × 2 (3004)
	1 × 2 (3004)	1 × 3/25° (4286)	1 × 3/25° (4286)
Large	1 × 3 (3622)	2 × 2 (3003)	1 × 4 (3010)
	2 × 2 (3003)	2 × 3 (3002)	2 × 2 (3003)
	1 × 8 (3008)	1 × 6 (3009)	2 × 4 (3001)

For each participant, one of the three target sets was in bright red, bright blue, and bright yellow, respectively. The Element ID of each target can be retrieved by adding the color code (bright red: 21, bright blue: 23, bright yellow: 24) to the respective Design ID mentioned in brackets. For example, 428623 for the bright blue ROOF TILE 1 × 3/25°.

#### Experimental procedure

Participants received written information that they had to search for a fixed number of LEGO® bricks (3, 4, or 5) with a defined color (blue, red, or green), pick them up and place them according to one of three instructions (collect, sort, pile). A trial started with playback of auditory instructions about the immediately upcoming task (e.g., “Collect five blue ones.”; translated from the German original). After completion of each trial, the experimenter placed the selected targets from the placing area back in the picking area. For a block consisting of nine trials, the LEGO® brick configuration in the picking area roughly stayed the same. After the block, the experimenter randomly distributed the same set of LEGO® bricks in a 5 × 8 3D grating on the picking area. The grating was then removed, and hence not visible to participants during search. Participants’ head positions were not fixed, they could move their heads freely. The viewing distance to the center of the stimuli arrangement was about 60 to 85 cm, depending on how tall the participant was. To familiarize themselves with the task, participants performed one practice block with nine trials, after which they could ask questions if they had any. Then, the main experiment started, which consisted of 81 trials. In every block, each placing instruction and each target color occurred three times in a random order. Search size was random but balanced across trials, ensuring that a specific combination of instruction (three placing instructions × three search sizes × three target colors) occurred three times throughout the main experiment.

### Quantification and statistical analysis

#### Pose estimation

While participants performed the task, their hand movements were recorded in a 1,440 by 1,080 pixels video at 200 frames per second. DeepLabCut^58^^,^^63^ (version 2.2.1), a tool for markerless pose estimation based on deep neural networks, was used to estimate the participants’ hand positions and subsequently extracted movement parameters. The analysis was conducted on a workstation running an Ubuntu 20.04.4 LTS 64-bit operating system with an Intel Core i9-9820X CPU (3.3 GHz) and an NVIDIA Geforce RTX 2080 SUPER GPU. Using Conda 4.12.0, an anaconda environment was created running Python 3.8.13. DeepLabCut 2.2.1 and Tensorflow 2.9.1 were installed. The full set of packages and package versions can be found in the OSF repository (https://osf.io/c938e/).

The fingertips as well as interphalangeal and metacarpophalangeal joints of thumb, index finger, and middle finger were labeled in 20 frames per video, using one video from each of 26 participants. Of the 520 labeled frames, 95% were used for training and the remaining 5% for validation. A ResNet-50-based neural network^64^^,^^65^ with default parameters was trained for 1,030,000 iterations. Validation showed a test error of 4.73 pixels (train: 2.3 pixels). The network was then used to analyze all videos from all participants. Each video resulted in an H5 file with X, Y coordinates for all tracked fingertips and joints of thumb, index finger, and middle finger for all frames and the corresponding likelihood score. The likelihood score lies between 0 and 1 and represents the confidence of the network in the estimated pose.

#### Data preprocessing

The X- and Y-trajectory data were preprocessed trial-wise with Python 3.7.3 using modules from Pandas 1.2.4, NumPy 1.21.4, and SciPy 1.7.3^60^ to estimate position and speed of the hand. All processing was limited to the data from the tracked metacarpophalangeal joint of the right index finger. This specific joint was well-trackable from the top-down view the setup provided. If the confidence of the network in the estimated pose (likelihood score) was below 0.7, the X, Y coordinates were linearly interpolated.

Movement data were segmented into reach and transport movements based on finding their start and end by multiplying different sources of information into a single, “objective function.”^25^ In this approach, different information sources are transformed into probability signals and then multiplied to obtain an objective function that maps high probability to points of interest. For example, the probability that a movement starts is high when the movement speed is low and the hand is near the starting position. For each trial, two objective functions were calculated: one for detecting the movement starts and one for detecting the movement ends in the movement sequence. To obtain an objective function, we integrated data from hand position, inverted speed, a speed threshold of 30 cm/frame, an acceleration threshold of 0.5 cm/frames^2^ that removed artefacts, and a likelihood threshold of 0.9. Speed was calculated as the pixelwise Euclidean distance between the Cartesian coordinates (x_i_, y_i_) and (x_i+1_, y_i+1_) over time (i.e., successive video frames). The resulting speed was then smoothed with a third-order Savitzky-Golay filter with window size of 59. Acceleration was computed as the gradient of smoothed speed, and again smoothed with the same Savitzky-Golay filter. In addition, shifted Gaussian (SD = 9) peaks were multiplied with the signal to down weight probabilities in ranges far from the points of interest.

To suppress irrelevant signals around the signal of interest, a Gaussian filter with filter size 9 was added 100 frames after each movement start to the movement end detection. The same Gaussian filter was added 100 frames before each movement end to the movement start detection. This suppresses activity related to the movement start when looking for the movement end, and vice versa. Peaks in the objective function that represent the movement start or end, respectively, were then detected using the find_peaks function of SciPy.^60^ For peak detection, a minimum distance of 50 frames (250 ms) between two consecutive movement starts or ends, respectively, was required. Finally, the detected number of movement starts and ends in a trial were required to equal the respective search size. If this check failed, the trial was discarded and not considered for further analysis. Meta data (participant number, experimental condition, trial number) were stored along with the calculated dependent variables of interest listed in the table Dependent Variables and How They Were Calculated. The dependent variables were calculated for each movement segment (first reach movement, first transport movement, second reach movement, and so forth).

#### Dependent variables and how they were calculated

**Table:** 

Dependent Variable (Unit)	Description
Movement duration (ms)	Number of frames in a movement, converted to milliseconds
Average movement speed (cm/s)	Mean value of the smoothed speed for each movement
Peak movement speed (cm/s)	Maximum value of the smoothed speed in a movement
Deceleration time (%)	Min-max normalized duration after peak movement speed
Movement distance (cm)	Sum of the Euclidean distances (pixels) between the hand position in two consecutive frames of a movement, converted to centimeters
Movement onset (ms)	Time between the end of the audio instructions and the detected start of the first reach movement

Four participants were excluded because they frequently started trials with a hand movement in the direction of their own body, which was outside the recorded area. Due to this, the metacarpophalangeal joint of their index finger could not be tracked, making a precise pose estimation for the first reach movement impossible.

Moreover, 368 trials from the reach movements and 280 trials from the transport movements were discarded during movement segmentation because the number of detected movements did not equal the respective search size. Another 69 trials were removed manually from both data frames for known technical or human errors. If more than 1/3 of participant’s trials were removed, we excluded the participant entirely from the analysis. Two participants were excluded based on this criterion, leaving 1637 trials for reach movement analysis, and 1731 trials for transport movement analysis out of the total 1944 trials from 24 participants.

#### General statistical analysis

JASP 0.19^59^ was used for statistical treatment of the data in a Bayesian framework. We report Bayes factors (BF), representing relative model comparisons between null hypotheses H0 and alternative hypotheses H1. Evidence for the alternative hypothesis H1 is reflected in a high BF_10_, speaking against the null hypothesis H0. On the other hand, evidence for the null hypothesis H0 is reflected in a high BF_01_, speaking against the alternative hypothesis H1. For data analysis with ANOVAs, effect analyses on matched models were used.^59^ Evidence for the presence (absence) of an effect is represented by an inclusion (exclusion) Bayes factor BF_incl_ (BF_excl_).^66^ Bayesian *t*-tests were performed as pairwise post hoc tests with uncorrected Bayes factors (BF_10, U_ or BF_01, U_).

For most statistical analyses of the dependent variables, the means of the movement data were first aggregated by participant, task, and search size (number of targets to be collected). The factors included in each Bayesian repeated measures ANOVA can be found in the statistical analysis tables of the dependent variables below.

Whenever proportions were extreme (not between 0.3 and 0.7), data were arcsine transformed to stabilize variance before calculating statistics.

All JASP^59^ statistical analysis files can be found in the OSF repository (https://osf.io/c938e/).

#### Statistical analysis of first transport deceleration time (%)

**Table:** Analysis of effects

Effects	P(incl)	P(excl)	P(incl|data)	P(excl|data)	BF_incl_
Task	0.500	0.500	1.000	2.300×10^−6^	434811.923

Compares models that contain the effect to equivalent models stripped of the effect. Higher-order interactions are excluded. Analysis suggested by Sebastiaan Mathôt.^59^

**Table:** Post hoc comparisons - task

		Prior odds	Posterior odds	BF_10, U_	error %
Collect	Sort	0.587	107.473	182.964	5.255×10^−8^
	Pile	0.587	4473.025	7614.943	1.701×10^−9^
Sort	Pile	0.587	2.897	4.932	2.663×10^−7^

The posterior odds have been corrected for multiple testing by fixing to 0.5 the prior probability that the null hypothesis holds across all comparisons.^67^ Individual comparisons are based on the default *t*-test with a Cauchy (0, r = 1/sqrt(2)) prior. The "U" in the Bayes factor denotes that it is uncorrected.

#### Statistical analysis of first reach deceleration time (%)

**Table:** Analysis of effects

Effects	P(incl)	P(excl)	P(incl|data)	P(excl|data)	BF_excl_
Task	0.400	0.400	0.106	0.892	8.404
Search Size	0.400	0.400	0.192	0.806	4.209
Task ✻ Search Size	0.200	0.200	0.002	0.021	9.554

Compares models that contain the effect to equivalent models stripped of the effect. Higher-order interactions are excluded. Analysis suggested by Sebastiaan Mathôt.^59^

**Table:** Post hoc comparisons - task

		Prior odds	Posterior odds	BF_01, U_	error %
Collect	Sort	1.702	7.175	4.214	0.068
	Pile	1.702	13.102	7.696	0.104
Sort	Pile	1.702	7.620	4.476	0.071

The posterior odds have been corrected for multiple testing by fixing to 0.5 the prior probability that the null hypothesis holds across all comparisons.^67^ Individual comparisons are based on the default *t*-test with a Cauchy (0, r = 1/sqrt(2)) prior. The "U" in the Bayes factor denotes that it is uncorrected.

#### Statistical analysis of average first reach speed

**Table:** Analysis of effects

Effects	P(incl)	P(excl)	P(incl|data)	P(excl|data)	BF_incl_
Task	0.400	0.400	0.856	0.141	6.072
Search Size	0.400	0.400	0.099	0.898	0.110
Task ✻ Search Size	0.200	0.200	0.003	0.085	0.032

Compares models that contain the effect to equivalent models stripped of the effect. Higher-order interactions are excluded. Analysis suggested by Sebastiaan Mathôt.^59^

**Table:** Post hoc comparisons - task

		Prior odds	Posterior odds	BF_10, U_	error %
Collect	Sort	1.702	0.097	0.165	0.088
	Pile	1.702	2.042	3.476	0.008
Sort	Pile	1.702	66.596	113.375	1.137×10^−8^

The posterior odds have been corrected for multiple testing by fixing to 0.5 the prior probability that the null hypothesis holds across all comparisons.^67^ Individual comparisons are based on the default *t*-test with a Cauchy (0, r = 1/sqrt(2)) prior. The "U" in the Bayes factor denotes that it is uncorrected.

#### Statistical analysis of first reach distance

**Table:** Analysis of effects

Effects	P(incl)	P(excl)	P(incl|data)	P(excl|data)	BF_incl_
Task	0.400	0.400	0.802	0.195	4.123
Search Size	0.400	0.400	0.104	0.893	0.116
Task ✻ Search Size	0.200	0.200	0.003	0.084	0.034

Compares models that contain the effect to equivalent models stripped of the effect. Higher-order interactions are excluded. Analysis suggested by Sebastiaan Mathôt.^59^

**Table:** Post hoc comparisons - task

		Prior odds	Posterior odds	BF_10, U_	error %
Collect	Sort	0.587	0.081	0.138	0.100
	Pile	0.587	1.971	3.355	0.009
Sort	Pile	0.587	21.056	35.846	4.828×10^−8^

The posterior odds have been corrected for multiple testing by fixing to 0.5 the prior probability that the null hypothesis holds across all comparisons.^67^ Individual comparisons are based on the default *t*-test with a Cauchy (0, r = 1/sqrt(2)) prior. The "U" in the Bayes factor denotes that it is uncorrected.

**Table:** Bayesian paired samples t-test

Measure 1		Measure 2	BF_+0_	error %
Reach Distance (cm) Pile big	-	Reach Distance (cm) Collect big	7.805	∼2.038×10^−4^
Reach Distance (cm) Pile big	-	Reach Distance (cm) Sort big	13.187	∼6.348×10^−4^

For all tests, the alternative hypothesis specifies that Measure 1 is greater than Measure 2. For example, Reach Distance (cm) Pile big is greater than Reach Distance (cm) Collect big.

**Table:** Bayesian paired samples t-test

Measure 1		Measure 2	BF_0+_	error %
Reach Distance (cm) Pile small	-	Reach Distance (cm) Collect small	8.834	∼0.082
Reach Distance (cm) Pile small	-	Reach Distance (cm) Sort small	6.172	∼7.206×10^−4^

For all tests, the alternative hypothesis specifies that Measure 1 is greater than Measure 2. For example, Reach Distance (cm) Pile small is greater than Reach Distance (cm) Collect small. Ten missing values for pile, and two for collect and sort because some participants never selected a small object first in those conditions.

#### Statistical analysis of reach movement onset

**Table:** Analysis of effects

Effects	P(incl)	P(excl)	P(incl|data)	P(excl|data)	BF_incl_
Task	0.400	0.400	0.203	0.525	0.387
Search Size	0.400	0.400	0.075	0.653	0.115
Task ✻ Search Size	0.200	0.200	0.272	0.021	13.012

Compares models that contain the effect to equivalent models stripped of the effect. Higher-order interactions are excluded. Analysis suggested by Sebastiaan Mathôt.^59^

#### Statistical analysis of first reach duration

**Table:** Analysis of effects

Effects	P(incl)	P(excl)	P(incl|data)	P(excl|data)	BF_incl_
Task	0.400	0.400	0.453	0.532	0.870
Search Size	0.400	0.400	0.264	0.736	0.350
Task ✻ Search Size	0.200	0.200	0.006	0.120	0.052

Compares models that contain the effect to equivalent models stripped of the effect. Higher-order interactions are excluded. Analysis suggested by Sebastiaan Mathôt.^59^

#### Statistical analysis of target size proportion

**Table:** Bayesian one sample t-test

	BF_10_	error %
Target Size Proportion (%) Collect big	1.457×10^8^	4.386×10^−11^
Target Size Proportion (%) Sort big	6.157×10^6^	1.180×10^−10^
Target Size Proportion (%) Pile big	2.959×10^7^	1.671×10^−10^

For all tests, the alternative hypothesis specifies that the population mean differs from 0.785, which corresponds to the arcsine-transformed expectation of 50%.

**Table:** Bayesian paired samples t-test

Measure 1		Measure 2	BF_+0_	error %
Target Size Proportion (%) Pile big	-	Target Size Proportion (%) Collect big	6.130	∼ 9.032×10^−5^
Target Size Proportion (%) Pile big	-	Target Size Proportion (%) Sort big	34.889	∼ 6.703×10^−4^

For all tests, the alternative hypothesis specifies that Measure 1 is greater than Measure 2. For example, Target Size Proportion (%) Pile big is greater than Target Size Proportion (%) Collect big.

#### Quantification of picking and placing activation

Heatmaps were created on the basis of difference images that encode what has changed between the first and last image of a video, which reflects where picking and placing actions occurred during a trial. To this end, images from start and end of each trial were loaded, converted to HSV (hue, saturation, value) representations and cropped to the relevant areas in the images (i.e., picking and placing area). Pixels in the image were set to maximum (255) if the saturation and value were larger than 50 in the original HSV image. This step removed the gray background, encoded where saturated objects (the LEGO® bricks) were present, and accounted for artefacts from slight lighting changes. From each of the two images, a new image was created with a Gaussian blur (radius = 5). The two blurred images were then subtracted from each other. Next, the values were normalized into the range of 0 % to 100 %, and another Gaussian blur (radius = 5) was applied for further smoothing. To remove artefacts, such as from slight lighting changes or objects that were only moved slightly, pixels with low values (below 150) were set to zero before applying a final smoothing (Gaussian blur, radius = 5). For each activation image, the resulting mean activations of the quadrants were stored in a list with the respective meta data for statistical analysis. The image processing was conducted with the Python module Pillow.

#### Statistical analysis of picking activation

**Table:** Analysis of effects

Effects	P(incl)	P(excl)	P(incl|data)	P(excl|data)	BF_incl_
Quadrant	0.263	0.263	0.983	2.107×10^−6^	466367.275
Task	0.263	0.263	0.024	0.975	0.025
Search Size	0.263	0.263	0.983	1.270×10^−12^	7.740×10^11^
Quadrant ✻ Task	0.263	0.263	5.584×10^−4^	0.024	0.023
Quadrant ✻ Search Size	0.263	0.263	0.017	0.983	0.017
Task ✻ Search Size	0.263	0.263	2.044×10^−4^	0.025	0.008
Quadrant ✻ Task ✻ Search Size	0.053	0.053	1.111×10^−6^	7.891×10^−8^	14.075

Compares models that contain the effect to equivalent models stripped of the effect. Higher-order interactions are excluded. Analysis suggested by Sebastiaan Mathôt.^59^

**Table:** Post hoc comparisons - quadrant

		Prior odds	Posterior odds	BF_10, U_	error %
Quadrant 1	Quadrant 2	0.414	5805.043	14014.614	1.378×10^−10^
	Quadrant 3	0.414	8.423×10^14^	2.033×10^15^	1.292×10^−17^
	Quadrant 4	0.414	7.100×10^15^	1.714×10^16^	1.294×10^−18^
Quadrant 2	Quadrant 3	0.414	39933.082	96406.989	1.832×10^−11^
	Quadrant 4	0.414	25.513	61.594	4.566×10^−4^
Quadrant 3	Quadrant 4	0.414	0.058	0.141	0.145

The posterior odds have been corrected for multiple testing by fixing to 0.5 the prior probability that the null hypothesis holds across all comparisons.^67^ Individual comparisons are based on the default *t*-test with a Cauchy (0, r = 1/sqrt(2)) prior. The "U" in the Bayes factor denotes that it is uncorrected.

**Table:** Post hoc comparisons - search size

		Prior odds	posterior odds	BF_10, U_	error %
3	4	0.414	1.542×10^7^	2.625×10^7^	6.323×10^−14^
	5	0.414	5.718×10^21^	9.735×10^21^	2.300×10^−24^
4	5	0.414	70447.748	119931.260	1.675×10^−11^

The posterior odds have been corrected for multiple testing by fixing to 0.5 the prior probability that the null hypothesis holds across all comparisons.^67^ Individual comparisons are based on the default *t*-test with a Cauchy (0, r = 1/sqrt(2)) prior. The "U" in the Bayes factor denotes that it is uncorrected.

#### Statistical analysis of placing activation

**Table:** Analysis of effects

Effects	P(incl)	P(excl)	P(incl|data)	P(excl|data)	BF_incl_
Quadrant	0.263	0.263	9.570×10^−25^	3.161×10^−44^	3.027×10^19^
Task	0.263	0.263	9.860×10^−23^	9.165×10^−25^	107.583
Search Size	0.263	0.263	0.009	2.608×10^−15^	3.584×10^12^
Quadrant ✻ Task	0.263	0.263	0.988	1.007×10^−22^	9.805×10^21^
Quadrant ✻ Search Size	0.263	0.263	0.978	0.010	102.563
Task ✻ Search Size	0.263	0.263	0.022	0.966	0.023
Quadrant ✻ Task ✻ Search Size	0.053	0.053	0.012	0.022	0.558

Compares models that contain the effect to equivalent models stripped of the effect. Higher-order interactions are excluded. Analysis suggested by Sebastiaan Mathôt.^59^

**Table:** Post hoc comparisons - quadrant

		Prior odds	Posterior odds	BF_10, U_	error %
Quadrant 1	Quadrant 2	0.414	0.075	0.181	0.115
	Quadrant 3	0.414	5.696×10^21^	1.375×10^22^	1.504×10^−25^
	Quadrant 4	0.414	3.427×10^40^	8.273×10^40^	3.379×10^−47^
Quadrant 2	Quadrant 3	0.414	4.445×10^32^	1.073×10^33^	7.264×10^−39^
	Quadrant 4	0.414	1.426×10^38^	3.444×10^38^	7.540×10^−45^
Quadrant 3	Quadrant 4	0.414	2.002×10^12^	4.834×10^12^	8.106×10^−20^

The posterior odds have been corrected for multiple testing by fixing to 0.5 the prior probability that the null hypothesis holds across all comparisons.^67^ Individual comparisons are based on the default *t*-test with a Cauchy (0, r = 1/sqrt(2)) prior. The "U" in the Bayes factor denotes that it is uncorrected.

**Table:** Post hoc comparisons - task

		Prior odds	Posterior odds	BF_10, U_	error %
Collect	Sort	0.587	0.039	0.066	0.310
	Pile	0.587	3457.181	5885.554	5.208×10^−6^
Sort	Pile	0.587	10.220	17.398	0.001

The posterior odds have been corrected for multiple testing by fixing to 0.5 the prior probability that the null hypothesis holds across all comparisons.^67^ Individual comparisons are based on the default *t*-test with a Cauchy (0, r = 1/sqrt(2)) prior. The "U" in the Bayes factor denotes that it is uncorrected.

**Table:** Post hoc comparisons - search size

		Prior odds	Posterior odds	BF_10, U_	error %
3	4	0.414	1.948×10^10^	3.316×10^10^	3.523×10^−17^
	5	0.414	5.609×10^23^	9.548×10^23^	1.607×10^−26^
4	5	0.414	2.060×10^8^	3.507×10^8^	4.236×10^−15^

The posterior odds have been corrected for multiple testing by fixing to 0.5 the prior probability that the null hypothesis holds across all comparisons.^67^ Individual comparisons are based on the default *t*-test with a Cauchy (0, r = 1/sqrt(2)) prior. The "U" in the Bayes factor denotes that it is uncorrected.

## Supplemental information

Table S1. LEGO Brick Name, Design ID, color, and Element ID by color of non-targets, related to STAR Methods
