# Supplementary material for: Picking the right piece: Action intentions shape visual search and action planning in human multi-target-foraging
Source: iScience. 2025 May 13;28(6):112656. doi: 10.1016/j.isci.2025.112656 (PMC12152353; doi:10.1016/j.isci.2025.112656)
Supplement: Table S1. LEGO Brick Name, Design ID, color, and Element ID by color of non-targets, related to STAR Methods [file mmc1.pdf]

iScience, Volume *28*

Supplemental information

Picking the right piece: Action intentions shape visual search and action planning in human multi-target-foraging

Danilo A. Kuhn, Jan Tünnermann, and Anna Schubö

Table S1. LEGO® Brick Name, Design ID, color, and Element ID by color of non-targets, related to STAR Methods.

Brick Name	Design ID	Color	Element ID by Color
PLATE 2X4	3020	Brick Yellow	4114309
ROOF TILE 1X3/25°	4286	Black	428626
BRICK 2X2	3003	Lavender	6099349
ROOF TILE 2X3/45°	3038	Medium Lavender	6036782
ROOF TILE 1X2/45°	3040	Bright Reddish Violet	4625626
BRICK 2X2	3003	Bright Orange	4153825
ROOF TILE 2X1X2	60481	Lavender	6331921
ROOF TILE 2X1X2	60481	White	4515370
ROOF TILE 4x2/45° INV.	4871	Medium Stone Grey	4211517
BRICK 2X4	3001	Reddish Brown	4211201
BRICK 2X4	3001	Dark Green	4106356
BRICK 2X3	3002	Dark Green	4109674
BRICK 1X3	3622	Dark Green	4109679
BRICK 2X3	3002	Reddish Brown	4216668
FLAT TILE 1X4	2431	Black	243126
BRICK 1X2	3004	Bright Yellowish Green	4164022
BRICK 1X1	3005	Bright Yellowish Green	4220634
BRICK 1X1	3005	White	300501
PLATE 1X6	3666	Dark Stone Grey	4211056
BRICK 1X2X5	35274	Transparent	6251285
RIM WIDE W.CROSS 30X20	56145	Black	4299389
JET ENGINE	4868	Dark Stone Grey	4579639
